# Graded water supply decouples vegetative growth, reproductive investment, and nitrogen balance in trimorphic *Atriplex aucheri*

**DOI:** 10.1186/s12870-026-09676-3

**Published:** 2026-08-03

**Authors:** Muhammad Rizwan Shoukat, Yunling Zhang, Kai Yan, Zeeshan Ali Buttar, Li Jiang

**Affiliations:** 1 State Key Laboratory of Desert and Oasis Ecology, Xinjiang Institute of Ecology and Geography, Chinese Academy of Sciences, Urumqi, 830011 China; 2 General Grassland Station of Xinjiang, Urumqi, 830049 China

**Keywords:** *Atriplex aucheri*, Seed heteromorphism, Reproductive allocation, Water availability, C:N:P stoichiometry, Bet-hedging, Halophyte

## Abstract

**Background:**

Water availability is a primary driver of plant performance in arid ecosystems, yet its effects on reproductive allocation, tissue stoichiometry, and ion homeostasis remain poorly resolved in heterocarpic desert annuals. *Atriplex aucheri* Moq. is a trimorphic halophyte of the Junggar Basin that produces three seed types with contrasting dormancy and dispersal. We grew *A. aucheri* under four graded water regimes (100, 150, 200, and 250 mL day^− 1^) in a greenhouse experiment testing how water supply affects shoot biomass, morph-specific seed production, shoot C: N:P stoichiometry, and inorganic ion composition.

**Results:**

Increasing water raised shoot dry weight up to 60.6% (*p* < 0.001), whereas total seed allocation did not increase proportionally and instead declined along a significant linear trend, decreasing by 35.2% from the 100 mL to the 250 mL treatment (linear contrast *p* = 0.022). The morphs differed in their responses. Type A seed production peaked at intermediate water (quadratic contrast *p* = 0.016), type B remained stable, and type C allocation declined monotonically. Shoot nitrogen fell under the two highest water levels (*p* < 0.05) and the C: N ratio rose up to 29.8% relative to the 150 mL treatment, consistent with growth-driven nitrogen dilution. Of six ions measured, only sulfate responded to treatment (*p* < 0.05), decreasing 45.9% from the 150 mL to the 250 mL level, while sodium stayed dominant (58 to 61% of measured cations). Piecewise structural equation modelling indicated that water influenced reproductive allocation more consistently through a size-mediated pathway (β = 0.74, *p* < 0.001) than through tissue nitrogen, although the path from shoot dry weight to allocation was not significant.

**Conclusions:**

Higher water supply promoted vegetative growth without a proportional increase in reproductive investment, altered shoot stoichiometry, and had limited effects on most measured shoot ions. These findings provide insight into the response of *Atriplex aucheri* to graded water supply under greenhouse conditions.

**Supplementary Information:**

The online version contains supplementary material available at https://doi.org/10.1186/s12870-026-09676-3.

## Background

Arid and semi-arid regions cover more than 40% of Earth’s land surface and support over 2.5 billion people, yet these dryland ecosystems face increasing vulnerability under climate change [1–4]. Precipitation regimes across global drylands are projected to become more variable, with altered seasonality and more frequent extreme rainfall events in the coming decades [5, 6]. Water availability is the primary driver of plant productivity in these systems, and even modest changes in rainfall amount or timing can trigger nonlinear shifts in vegetation cover and ecosystem functioning [7, 8]. Predicting dryland vegetation dynamics under future climate scenarios therefore requires a mechanistic understanding of how individual species adjust their growth and reproductive strategies in response to water supply [9].

Life-history theory predicts that resource allocation to reproduction entails costs to vegetative growth, survival, or future reproduction [10, 11]. In annual plants, the partitioning of assimilated carbon between vegetative structures and reproductive output is a critical determinant of fitness [12, 13]. When resource availability increases, plants may channel additional carbon into vegetative growth rather than proportionally increasing seed production, resulting in declining reproductive allocation [14, 15]. This pattern has been documented in dryland systems where water addition promoted biomass accumulation without a corresponding increase in seed yield [16, 17]. Whether desert annuals with heteromorphic seed systems follow the same trade-off has not been tested.

Seed heteromorphism, the production of two or more morphologically distinct seed types by a single individual, occurs in over 200 angiosperm species and is especially prevalent in the Chenopodiaceae [18, 19]. Different seed morphs typically differ in size, dormancy, dispersal capacity, and germination requirements, and these differences function as a diversified bet-hedging strategy that spreads reproductive risk across time and space [20, 21]. The genus *Atriplex* provides well-studied examples; *A. sagittata* produces three fruit types differing in dormancy and dispersal [22, 23], while *A. centralasiatica* produces three diaspore types with distinct germination and seedbank persistence strategies in temperate salt deserts [24, 25]. Maternal environmental conditions, including salinity, nutrients, and water supply, can shift the proportional allocation to each morph, potentially reprogramming the bet-hedging portfolio of subsequent generations [26–29].

Water availability also influences the elemental composition of plant tissues. Ecological stoichiometry frames the balance among carbon (C), nitrogen (N), and phosphorus (P) as an indicator of nutritional status and growth strategy [30, 31]. When biomass increases rapidly without commensurate nutrient uptake, tissue N concentration declines, a phenomenon termed the nitrogen dilution effect [32, 33]. This pattern has been reported in Central Asian desert species [34, 35] but not in heterocarpic xerophytes. For halophytes in the Chenopodiaceae, shoot ion composition is equally informative, because Na^+^ accumulation drives osmotic adjustment through vacuolar sequestration and salt bladder function [36–38], yet how graded water supply modifies ion profiles in xerophytic chenopods has received limited attention [39, 40]. *Atriplex aucheri* Moq. is a heterocarpic desert annual confined to the Junggar Basin and Gobi desert margins, colonizing saline substrates with annual precipitation below 250 mm [41]. A single plant produces three seed types (A, B, and C) that differ in size, colour, dormancy, and dispersal, and that play different roles in population persistence [29, 34, 42]. These ecological and reproductive characteristics make *A. aucheri* an appropriate model for examining how water availability influences vegetative growth, reproductive allocation, and bet-hedging strategies in desert annuals [29, 34, 42]. Earlier studies examined how maternal water conditions shape seed traits [34] and transgenerational growth responses [42], but employed only two water levels and did not simultaneously assess vegetative growth, morph-specific seed allocation, stoichiometry, and ion content across a water gradient. Beyond its ecological role, *A. aucheri* is used as a fodder species, and *Atriplex* more broadly is valued for forage production and for revegetation and reclamation of salt-affected and degraded land in arid and semiarid regions [34, 43, 44]. Clarifying how water availability partitions resources between vegetative biomass and seed output in this species is therefore relevant both to its persistence in desert habitats and to its use in saline-land restoration and forage supply. Although the dormancy and dispersal differences among the three seed morphs have been documented, how water availability regulates the relative investment in each morph has not been quantified in this species. The present study addresses this gap by testing how graded water supply shapes morph-specific seed production alongside vegetative growth, tissue stoichiometry, and ion balance.

The present study aimed to address this gap by growing *A. aucheri* under a graded water supply in a greenhouse experiment. We examined (1) how increasing water availability affects shoot biomass and the allocation to each of the three heteromorphic seed types, and (2) how water supply modifies shoot C: N:P stoichiometry and inorganic ion content (Cl^−^, SO_4_^2−^, Ca^2+^, Mg^2+^, K^+^, and Na^+^). We hypothesized that excess water would promote vegetative growth but reduce proportional reproductive investment, and that the three seed types would show differential responses reflecting their distinct ecological roles in the bet-hedging strategy of this desert annual.

## Materials and methods

### Study species and seed collection

*Atriplex aucheri* Moq. (Chenopodiaceae, included in Amaranthaceae) is an early successional annual xerophyte distributed across the Gobi desert, arid valleys, and wasteland of Central Asia [41]. The species produces three morphologically distinct seed types on a single plant [19, 34, 42]. Type A seeds are small and black, borne in female ebracteate flowers with a 5-lobed perianth. Type B seeds are medium-sized and black, produced by female bracteolate flowers and covered by extended bracteoles. Type C seeds are large and brown, also produced by female bracteolate flowers and enclosed in bracteoles. Type C seeds are non-dormant with a germination percentage typically ≥ 95%, whereas type A and type B seeds exhibit non-deep physiological dormancy and low germination percentages [34, 42].

Freshly matured fruits of *A. aucheri* were collected from a natural population in the hilly desert on the southern edge of the Junggar Basin in Xinjiang, China (43°46′N, 87°34′E), near the Fukang Desert Ecosystem Observation and Experiment Station of the Chinese Academy of Sciences (44°17′26″N, 87°55′58″E; 460 m a.s.l.) in October 2022. The sampling area has a typical temperate continental arid climate, with mean annual temperature of 6.6 °C, annual precipitation of approximately 160 mm, and annual potential evaporation exceeding 2000 mm [45, 46]. Seeds were separated from dried plant material, sorted into type A (small black), type B (medium-sized black), and type C (large brown), and stored at room temperature (19–25 °C) until use.

### Pot experiment

The experiment was conducted in a greenhouse (28 ± 3 °C day/18 ± 3 °C night, natural light conditions) at the Fukang Station. Approximately 20 type C seeds were sown at a soil depth of 2 mm into individual plastic pots (17 cm deep × 16 cm diameter) filled with 2 L of a loam soil and vermiculite mixture (3:1 v/v) on 10 April 2023. Seedlings were thinned to one per pot 20 days after sowing, and only seedlings of similar height were retained to reduce variation in initial size. Each pot therefore contained a single plant, which constituted the experimental unit; all seed and biomass measurements are expressed on a per-plant basis.

The experiment followed a randomized complete block design (RCBD) with seven blocks. Each block contained four pots, one assigned to each of four water treatments: 100, 150, 200, and 250 mL day^− 1^, yielding a total of 28 experimental units [42]. The lowest level (100 mL day^− 1^) approximated the baseline water supply under which *A. aucheri* completes its life cycle in cultivation, while the three higher levels represented progressively increasing water availability. Each pot received 1.2 g of slow-release granular fertilizer (Osmocote 301, Scotts, Marysville, OH, USA; 15 N:11P:13 K:2Mg) once before sowing as basal fertilizer. Water treatments were initiated 4 weeks after sowing and maintained daily until harvest. The experiment was completed on 14 October 2023, after which shoots and offspring seeds (F_1_) were harvested.

### Shoot dry weight and seed traits

After harvesting, shoots were oven-dried at 60 °C until constant weight and weighed to determine shoot dry weight per plant [42]. The number of seeds of each type (A, B, and C) on each plant was counted after detachment and cleaning. Seed yield (g) of each type per plant was measured using a microbalance (Chyo Balance JPN-200 W, Chyo Balance Corp., Japan). Seed allocation (%) for each type was calculated as [28, 42]:

1 $$\:Seed\:allocation=\frac{\mathrm{S}\mathrm{e}\mathrm{e}\mathrm{d}\:\mathrm{y}\mathrm{i}\mathrm{e}\mathrm{l}\mathrm{d}\:\mathrm{o}\mathrm{f}\:\mathrm{t}\mathrm{y}\mathrm{p}\mathrm{e}}{\mathrm{s}\mathrm{h}\mathrm{o}\mathrm{o}\mathrm{t}\:\mathrm{d}\mathrm{r}\mathrm{y}\:\mathrm{w}\mathrm{e}\mathrm{i}\mathrm{g}\mathrm{h}\mathrm{t}}\times\:100$$

Total seed number, total seed yield, and total seed allocation were calculated as the sum across the three seed types.

### Carbon, nitrogen, and phosphorus content

Dried shoot materials were ground in a ball mill and passed through a 0.25 mm sieve. The ball mill was cleaned between each sample to prevent cross-contamination. Shoot carbon (C) and nitrogen (N) contents were determined using an elemental analyzer (FLASHEA 1112 Series CNS Analyzer, Thermo Fisher Scientific, Waltham, MA, USA) [42]. Shoot phosphorus (P) content was determined colorimetrically using the molybdenum blue–ascorbic acid method [47] after digestion of ground plant material with HNO_3_–H_2_O_2_ [48]. The C: N, C: P, and N: P mass ratios were calculated from the measured elemental concentrations. All elemental concentrations are expressed in g kg^− 1^ dry weight.

### Shoot ion content

Six inorganic ions were measured in the dried shoot tissue, chloride (Cl^−^), sulfate (SO_4_^2−^), calcium (Ca^2+^), magnesium (Mg^2+^), potassium (K^+^), and sodium (Na^+^). After grinding the dried plant samples, the salt ion content in the aerial parts was determined. The concentrations of Na^+^, K^+^, Ca^2+^, and Mg^2+^ were determined by inductively coupled plasma mass spectrometry (ICP-MS; Perkin Elmer Sciex DRC II, PerkinElmer, Waltham, MA, USA). The contents of Cl^−^ and SO_4_^2−^ were determined by ion chromatography. All ion concentrations are expressed in g kg^− 1^ dry weight.

### Statistical analysis

Data were analyzed using a two-way analysis of variance (ANOVA) following the RCBD, with water treatment (four levels: 100, 150, 200, and 250 mL day^− 1^) as a fixed factor and block (seven blocks) as a random factor. Where the overall treatment effect was significant (α = 0.05), pairwise comparisons among water treatments were performed using Fisher’s protected least significant difference (LSD) test. Fisher’s protected LSD provides adequate Type I error control when the omnibus F-test is significant and the number of treatment comparisons is small (four treatments, and six pairwise comparisons) [49]. Residual normality was assessed by the Shapiro–Wilk test [50] and homogeneity of variances by Levene’s test. Because 25 response variables were analyzed separately, marginal *p*-values (0.01 < *p* < 0.05) are interpreted with caution. Because water was applied as a graded, equally spaced treatment, orthogonal polynomial contrasts (linear and quadratic) were fitted across the four water levels within the RCBD model to test the shape of the dose-response relationship for variables where directional trends were of interest. A significant linear contrast was interpreted as a monotonic increase or decrease, and a significant quadratic contrast as an intermediate optimum. All univariate analyses were conducted using Python 3.11 with the SciPy 1.17 library [51] and the statsmodels 0.14 package [52], and data are presented as means ± standard error (SE; *n* = 7).

Multivariate relationships among all 25 measured variables were explored using RDA, with water treatment as the constraining factor and block as a conditioning variable. All response variables were standardized to zero mean and unit variance prior to analysis. The significance of the treatment effect in multivariate space was tested by permutational multivariate analysis of variance (PERMANOVA) with 9999 permutations of treatment labels within the residualized space after removing block effects [53]. Pearson correlation coefficients among all 25 response variables were computed on block-residualized data, and *p*-values were adjusted using the Benjamini–Hochberg false discovery rate (FDR) correction [54].

To visualize compositional shifts in seed morph proportions, the relative contributions of the three seed types (by number) were plotted on a ternary diagram. Seed proportions were centered log-ratio (CLR) transformed to account for the unit-sum constraint [55], and treatment differences in compositional space were tested by PERMANOVA on the Aitchison distance matrix with 9999 permutations. A piecewise structural equation model (pSEM) was constructed to disentangle the direct and indirect pathways through which water availability affects seed allocation [56, 57]. In addition, the relative contribution of each seed morph to total seed output (the percentage of each type by number and by yield) was calculated per plant and analyzed with the same RCBD model to test whether water supply altered the reproductive portfolio independently of changes in absolute seed output. The model tested two competing pathways, one size-mediated, in which water affects seed allocation and total seed weight indirectly through shoot dry weight, and one nutrient-mediated, in which water affects seed allocation indirectly through shoot N content. The seed allocation node was modelled as a multiple regression with both shoot dry weight and N content as simultaneous predictors, controlling for confounding between the two variables. Model fit was evaluated using Fisher’s C statistic, where *p* > 0.05 indicates adequate fit [56]. All multivariate analyses were performed in Python 3.11 using NumPy [58], SciPy [51], scikit-learn [59], and statsmodels [52]. All figures were prepared using matplotlib 3.8 [60] in Python.

## Results

### Shoot dry weight

Two complementary analyses are reported throughout the Results. The first is a categorical ANOVA that compares the four water levels as discrete treatments, and the second is a set of orthogonal polynomial contrasts that test for continuous linear or quadratic trends across the graded gradient. Because the contrasts pool information along the dose axis, they can detect directional responses that the pairwise comparisons, with their lower power at *n* = 7, do not resolve; the two therefore need not agree for every variable.

Of the 25 measured variables, five responded significantly to water supply. Shoot dry weight increased with water availability (*p* < 0.001), whereas shoot nitrogen declined at the two highest levels (*p* = 0.024) and the C: N ratio rose correspondingly (*p* = 0.022). The N: P ratio also declined (*p* = 0.041), and among the six ions only sulfate (SO_4_^2−^) responded to treatment (*p* = 0.038). The remaining seed, stoichiometric, and ion variables showed no significant categorical treatment effect, although several followed significant linear or quadratic trends across the graded gradient (Table 1).

**Table 1:** Effects of water treatment on growth, seed traits, stoichiometric traits, and ion content in *Atriplex aucheri* based on RCBD ANOVA

Variable	F	*p*	*p* (linear)	*p* (quadratic)
Shoot dry weight (g plant^− 1^)	11.010	< 0.001 ***	< 0.001	0.535
Seed number of type A	3.134	0.051 ns	0.143	0.016
Seed number of type B	1.809	0.182 ns	0.526	0.342
Seed number of type C	1.268	0.315 ns	0.175	0.270
Total seed number	2.247	0.118 ns	0.383	0.072
Seed yield of type A (g)	1.985	0.152 ns	0.467	0.036
Seed yield of type B (g)	0.203	0.893 ns	0.534	0.692
Seed yield of type C (g)	0.684	0.573 ns	0.192	0.569
Total seed yield (g)	0.980	0.424 ns	0.922	0.117
Seed allocation of type A (%)	1.534	0.240 ns	0.315	0.070
Seed allocation of type B (%)	0.722	0.552 ns	0.232	0.462
Seed allocation of type C (%)	2.638	0.081 ns	0.009	0.916
Total seed allocation (%)	2.565	0.087 ns	0.022	0.221
C content (g kg^− 1^)	0.174	0.913 ns	0.921	0.862
N content (g kg^− 1^)	4.012	0.024 *	0.016	0.671
P content (g kg^− 1^)	0.377	0.771 ns	0.992	0.332
C: N ratio	4.109	0.022 *	0.011	0.752
C: P ratio	0.294	0.829 ns	0.733	0.401
N: P ratio	3.377	0.041 *	0.010	0.581
Cl⁻ content (g kg^− 1^)	2.884	0.064 ns	0.036	0.593
SO_4_^2−^ content (g kg^− 1^)	3.463	0.038 *	0.019	0.050
Ca^2+^ content (g kg^− 1^)	2.226	0.120 ns	0.073	0.871
Mg^2+^ content (g kg^− 1^)	1.948	0.158 ns	0.603	0.027
K^+^ content (g kg^− 1^)	2.739	0.074 ns	0.051	0.229
Na^+^ content (g kg^− 1^)	1.942	0.159 ns	0.035	0.532

Note: *** *p* < 0.001; ** *p* < 0.01; * *p* < 0.05; ns = not significant. Means separations are shown in the figures. The 25 response variables entered into the redundancy analysis, PERMANOVA, and Pearson correlation analyses comprise shoot dry weight; the seed number, yield, and allocation of the three morphs and their totals; C, N, and P content and the C: N, C: P, and N: P ratios; and the six measured ions (Cl^−^, SO_4_^2−^, Ca^2+^, Mg^2+^, K^+^, and Na^+^). The quadratic contrast for SO_4_^2−^ (*p* = 0.050) is treated as marginal and not significant under the *p* < 0.05 criterion

**Fig. 1:**
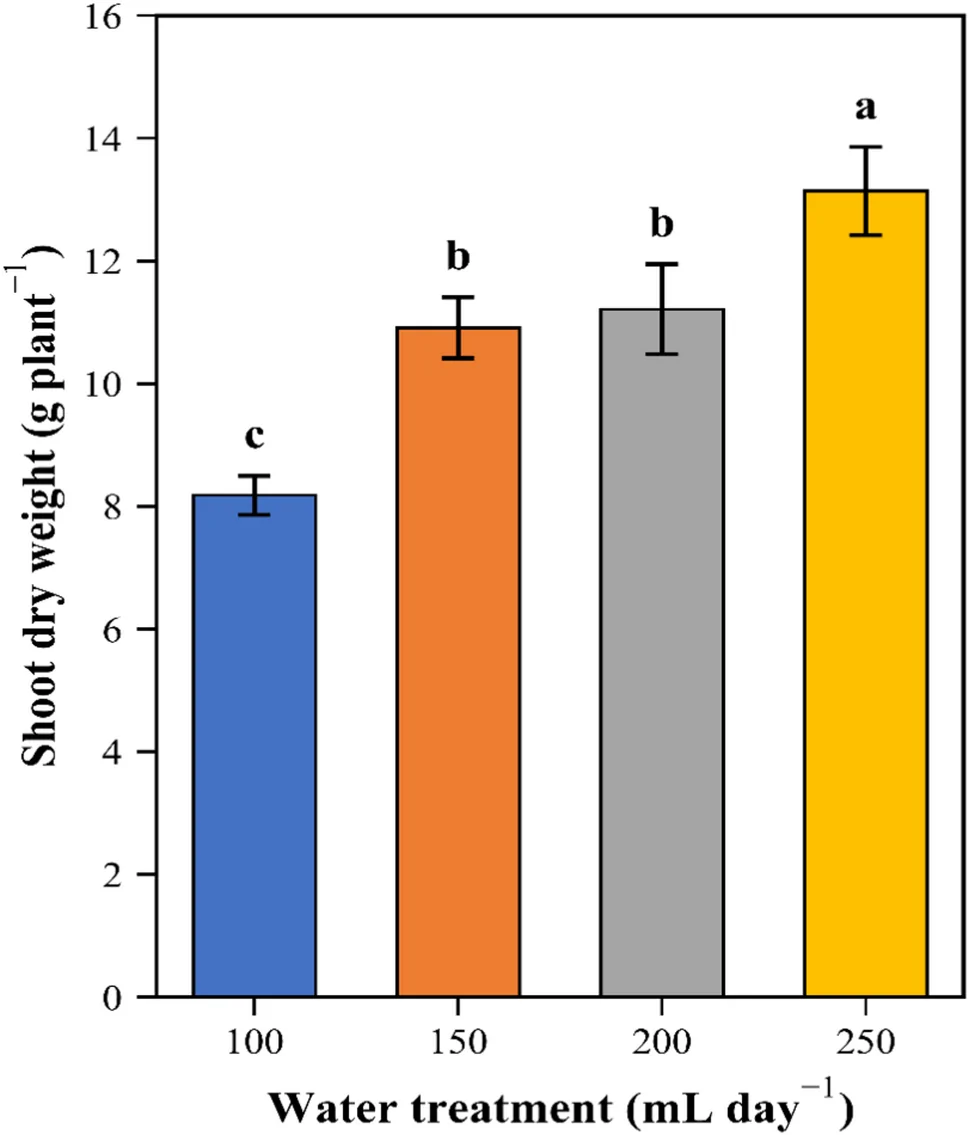
Effects of water treatment on shoot dry weight of *Atriplex aucheri*. Plants were grown under four water levels (100, 150, 200, and 250 mL day^− 1^) in a randomized complete block design with seven blocks. Data are presented as mean ± SE (*n* = 7). Different lowercase letters above bars indicate significant differences among treatments (*p* < 0.05; Fisher’s protected LSD test following RCBD ANOVA)

Water treatment had a highly significant effect on shoot dry weight of *A. aucheri* (*p* < 0.001; Table 1; Fig. 1). Shoot dry weight increased progressively with water availability, reaching the highest value under the 250 mL treatment, which represented a 60.6% increase over the 100 mL treatment. Plants receiving 100 mL water produced significantly lower biomass than those under all other treatments (*p* < 0.05). The 250 mL treatment also yielded significantly greater shoot dry weight than the 150 mL and 200 mL treatments, which did not differ from each other (Fig. 1).

### Seed number

**Fig. 2:**
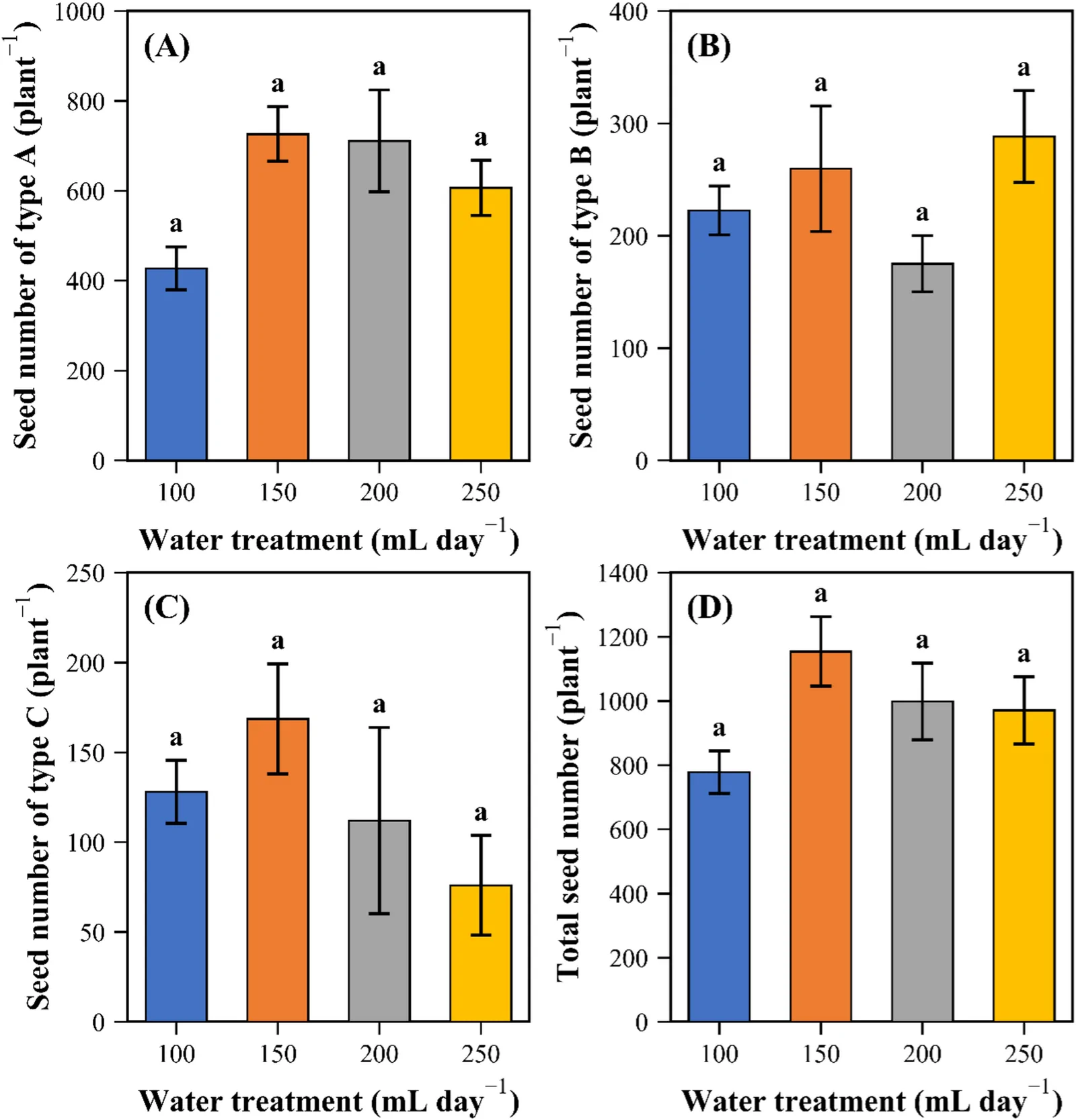
Seed number per plant of *Atriplex aucheri* under four water treatments. **A **Type A seeds (ebracteate, dormant). **B **Type B seeds (bracteolate, dormant). **C **Type C seeds (bracteolate, non-dormant). **D **Total seed number. Data are presented as mean ± SE (*n* = 7). Different lowercase letters above bars indicate significant differences among treatments (*p* < 0.05; Fisher’s protected LSD test following RCBD ANOVA)

No individual seed type differed significantly in number among water treatments, but type A seed number followed a significant quadratic trend, peaking at intermediate water (Table 1). The number of type A seeds (ebracteate, dormant) showed the strongest response, with a marginally non-significant overall treatment effect (*p* = 0.051; Table 1). The 150 mL treatment produced the highest number of type A seeds, 70.0% more than the 100 mL treatment, although pairwise comparisons did not detect significant differences among individual treatments (Fig. 2A). Consistent with this peak at intermediate water, the number of type A seeds followed a significant quadratic trend across the water gradient (quadratic contrast *p* = 0.016; Table 1), indicating an intermediate optimum rather than a monotonic response.

The numbers of type B seeds (bracteolate, dormant), type C seeds (bracteolate, non-dormant), and total seeds were also not significantly affected by water treatment (*p* > 0.05 for all; Table 1; Fig. 2B–D). Type A seeds constituted the largest proportion of total seed output across all treatments, followed by type B and type C. Total seed number under the 150 mL treatment was 48.4% higher than under the 100 mL treatment, but this difference did not reach statistical significance. Type C and total seed numbers varied most under the 200 mL treatment. Across the gradient, the type A quadratic and type C linear contrasts (Table 1) were the clearest directional signals in seed output.

### Seed yield

**Fig. 3:**
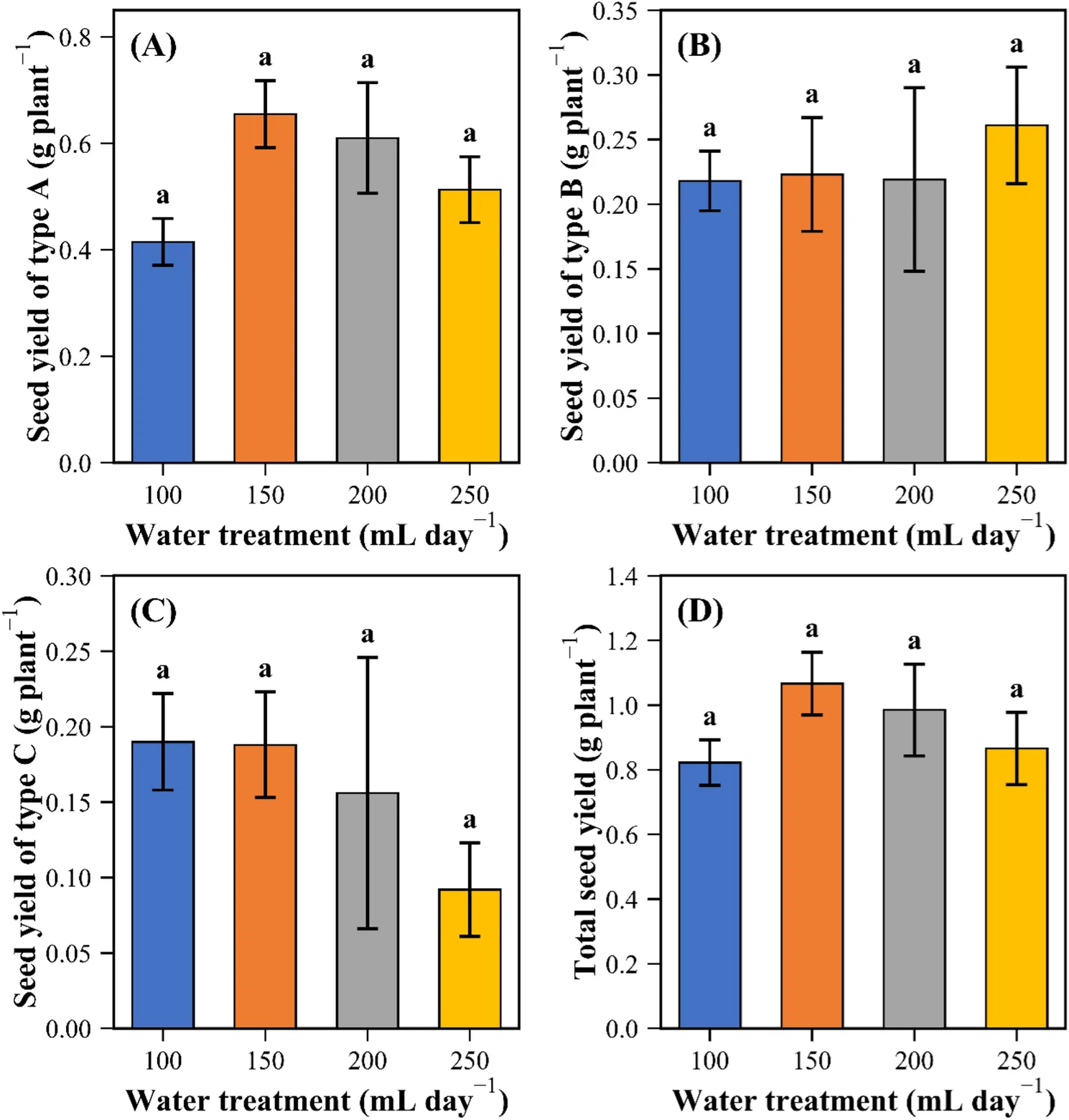
Seed yield (g plant^− 1^) of *Atriplex aucheri* across four water treatments. **A **Type A seeds (ebracteate, dormant). **B **Type B seeds (bracteolate, dormant). **C **Type C seeds (bracteolate, non-dormant). **D **Total seed yield. Data are presented as mean ± SE (*n* = 7). Different lowercase letters above bars indicate significant differences among treatments (*p* < 0.05; Fisher’s protected LSD test following RCBD ANOVA)

Seed yield (total seed mass per type per plant) showed no significant response to water treatment for any individual seed type or in total (*p* > 0.05; Table 1). The highest seed yield for type A was recorded under the 150 mL treatment, 54.8% greater than the 100 mL treatment, though this difference was not statistically significant (Fig. 3A). Seed yield of type B remained remarkably stable across all four treatments (Fig. 3B). Type C seed yield showed a declining pattern with increasing water, decreasing by 52.6% from the 100 mL to the 250 mL treatment, but this trend was not statistically significant (Fig. 3C). Total seed yield was highest under the 150 mL treatment and lowest under the 100 mL treatment, with no significant differences detected among treatments (Fig. 3D).

### Seed allocation

**Fig. 4:**
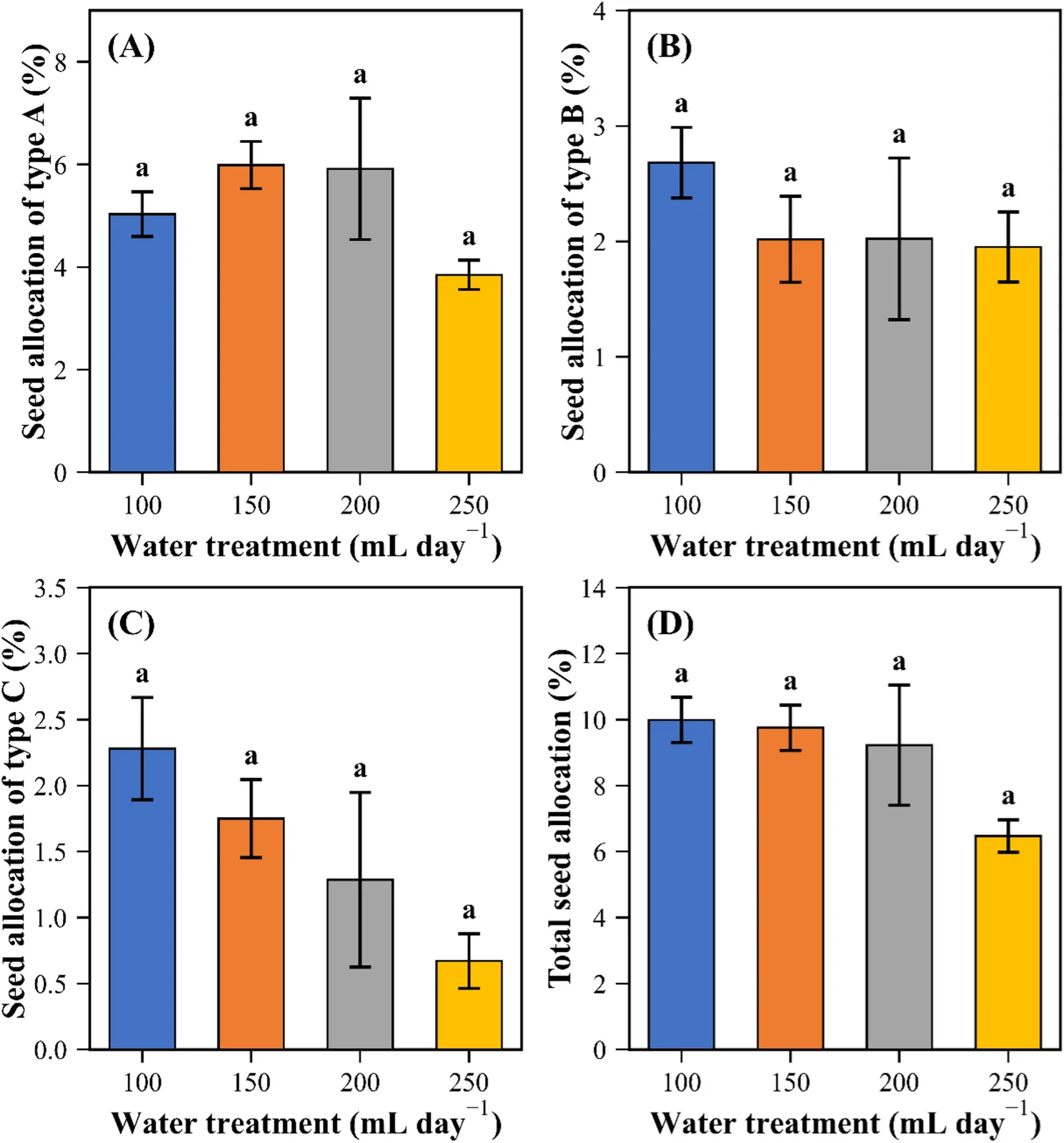
Seed allocation, expressed as seed yield relative to shoot dry weight (%), in *Atriplex aucheri* under four water treatments. **A** Type A seeds (ebracteate, dormant). **B **Type B seeds (bracteolate, dormant). **C **Type C seeds (bracteolate, non-dormant). **D **Total seed allocation. Data are presented as mean ± SE (*n* = 7). Different lowercase letters above bars indicate significant differences among treatments (*p* < 0.05; Fisher’s protected LSD test following RCBD ANOVA)

Seed allocation (seed yield as a percentage of shoot dry weight) was unaffected by water treatment for any individual seed type or in total (*p* > 0.05; Table 1). Allocation to type A seeds was highest under the 150 mL treatment and lowest under the 250 mL treatment (Fig. 4A). Allocation to type B seeds remained relatively constant across treatments (Fig. 4B). When water was treated as a graded variable, however, total seed allocation and type C allocation each declined along a significant linear trend (linear contrast *p* = 0.022 and *p* = 0.009, respectively; Table 1). Allocation to type C seeds decreased by 70.6% from the 100 mL to the 250 mL treatment. Although the categorical treatment effect was not significant, the decline across the graded water treatments was supported by a significant linear contrast (*p* = 0.009; Table 1) (Fig. 4C). Total seed allocation showed a similar declining pattern, with a 35.2% reduction from the 100 mL to the 250 mL treatment (Fig. 4D). The three seed types differed in their responses to water addition; type A allocation peaked at intermediate water (150 mL), type B allocation was insensitive to water level, and type C allocation declined monotonically with increasing water.

The relative reproductive portfolio showed that the proportional contribution of each morph to total seed yield did not change significantly with water supply, indicating a stable yield-based seed portfolio. By number, however, the relative contribution of type B varied significantly among treatments (*p* = 0.022), and that of type C declined along a significant linear trend (*p* = 0.033; Fig. S1).

### Shoot C, N, P content and stoichiometric ratios

**Fig. 5:**
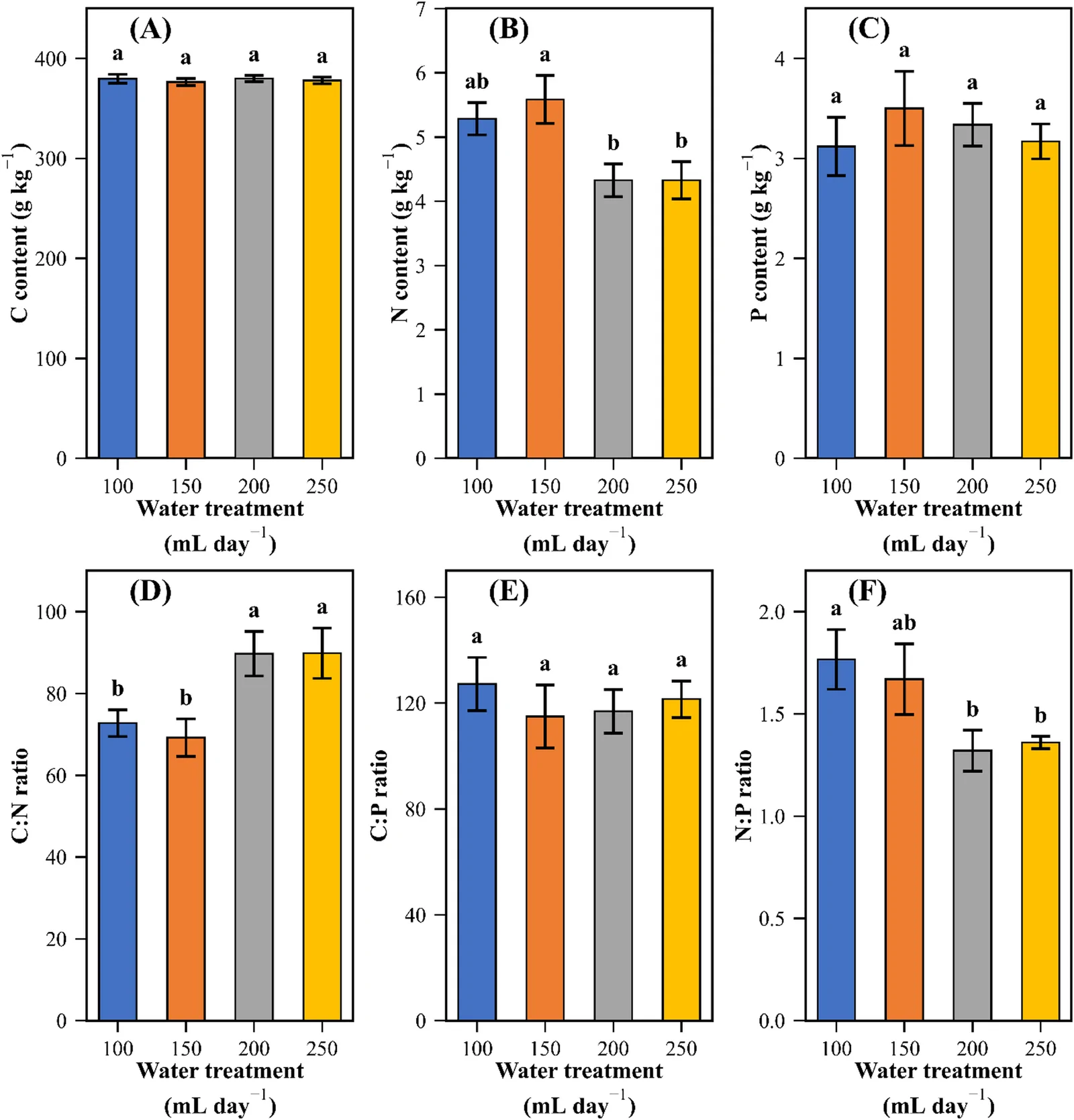
Shoot carbon, nitrogen, and phosphorus content and stoichiometric ratios of *Atriplex aucheri* under four water treatments. **A **Carbon content (g kg^− 1^). **B **Nitrogen content (g kg^− 1^). **C **Phosphorus content (g kg^− 1^). **D **C: N ratio. **E** C: P ratio. **F **N: P ratio. Data are presented as mean ± SE (*n* = 7). Different lowercase letters above bars indicate significant differences among treatments (*p* < 0.05; Fisher’s protected LSD test following RCBD ANOVA)

Shoot C content remained stable across all water treatments and showed no significant response (Table 1; Fig. 5A). Shoot N content was significantly influenced by water treatment (*p* < 0.05; Table 1). The 150 mL treatment had significantly higher N content than the 200 mL and 250 mL treatments (*p* < 0.05; Fig. 5B). The 100 mL treatment was intermediate and did not differ significantly from either the 150 mL or the 200/250 mL treatments. N content declined by 22.5% from the 150 mL to the 200/250 mL treatments. Shoot P content did not differ significantly among treatments (Table 1; Fig. 5C).

The C: N ratio was also significantly affected by water treatment (*p* < 0.05; Table 1). Plants under the 200 mL and 250 mL treatments exhibited significantly higher C: N ratios than those under the 100 mL and 150 mL treatments (*p* < 0.05; Fig. 5D), with increases of 29.6% and 29.8% relative to the 150 mL treatment, respectively. The two high-water treatments did not differ from each other. The C: P ratio did not differ significantly among treatments (Table 1; Fig. 5E). Water treatment had a significant overall effect on N: P ratio (*p* < 0.05; Table 1), with the 100 mL treatment having a significantly higher N: P ratio than the 200 mL and 250 mL treatments (*p* < 0.05; Fig. 5F). The 150 mL treatment was intermediate and did not differ significantly from either the 100 mL or the 200/250 mL treatments. The N: P ratio decreased by 25.4% from the 100 mL to the 200 mL treatment.

### Shoot ion content

**Fig. 6:**
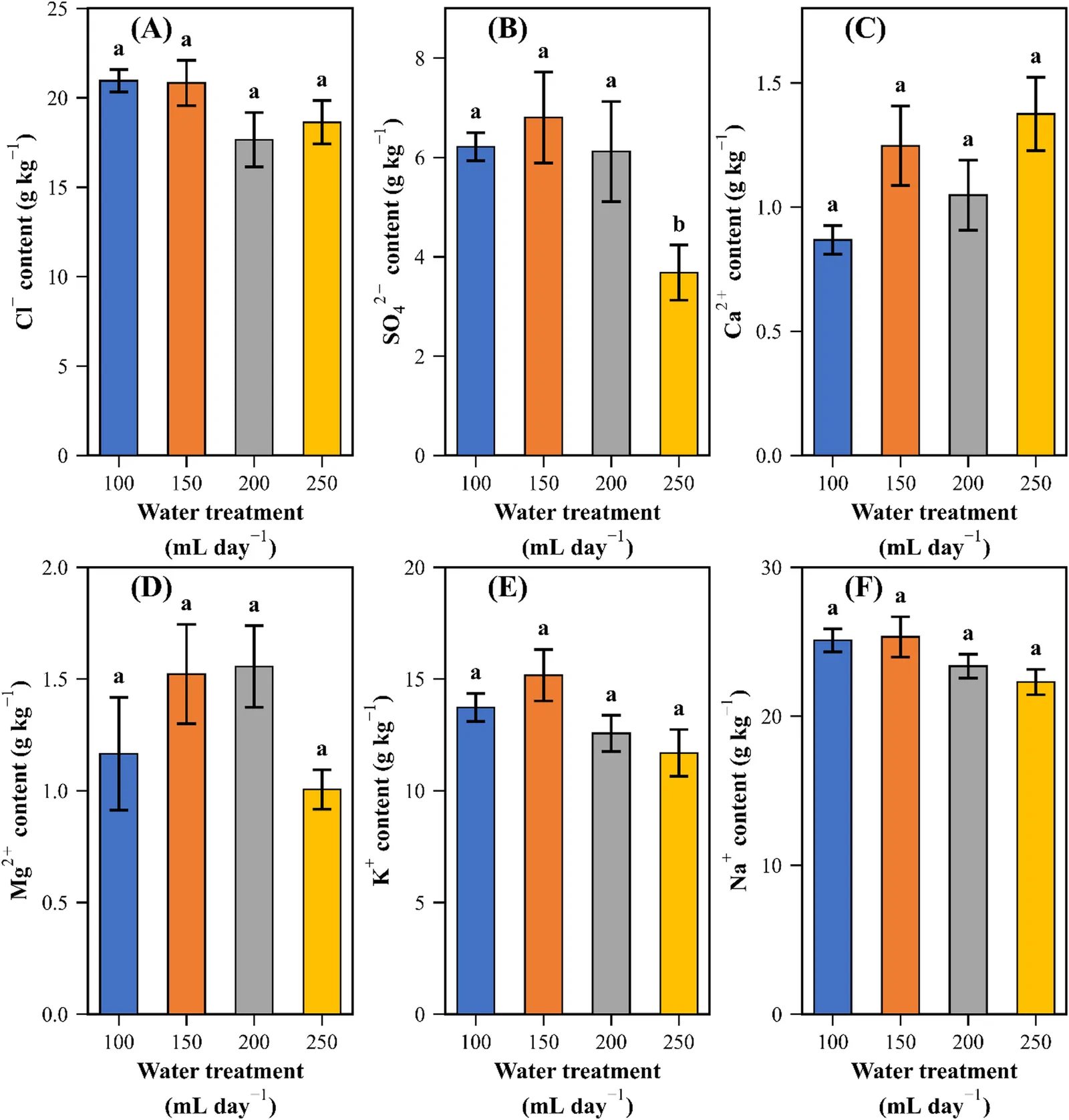
Shoot ion content (g kg^− 1^) of *Atriplex aucheri* in response to water treatment. **A **Chloride, Cl^−^. **B **Sulfate, SO_4_^2−^. **C **Calcium, Ca^2+^. **D **Magnesium, Mg^2+^. **E **Potassium, K^+^. **F **Sodium, Na^+^. Data are presented as mean ± SE (*n* = 7). Different lowercase letters above bars indicate significant differences among treatments (*p* < 0.05; Fisher’s protected LSD test following RCBD ANOVA)

Among the six ions analyzed (Cl^−^, SO_4_^2−^, Ca^2+^, Mg^2+^, K^+^, and Na^+^), only SO_4_^2−^ content was significantly affected by water treatment (*p* < 0.05; Table 1). Shoot Cl^−^ content showed a declining trend with increasing water, decreasing by 15.7% from the 100 mL to the 200 mL treatment, but the differences were not statistically significant (Table 1; Fig. 6A). The highest SO_4_^2−^ concentration was recorded under the 150 mL treatment, which was significantly higher than the 250 mL treatment, while the 100 mL and 200 mL treatments were intermediate (Fig. 6B). SO_4_^2−^ content under the 250 mL treatment was 45.9% lower than under the 150 mL treatment.

Ca^2+^ and Mg^2+^ contents did not differ significantly among treatments (Table 1; Fig. 6C, D). K^+^ content was highest under the 150 mL treatment but did not differ significantly among treatments (Table 1; Fig. 6E). Na^+^ content decreased from the 150 mL to the 250 mL treatment without reaching significance (Table 1; Fig. 6F). Across all water treatments, Na^+^ was the dominant cation, accounting for 58–61% of total measured cation content, followed by K^+^ (32–35%).

### Multivariate integration of growth, reproduction, and chemical responses

**Fig. 7:**
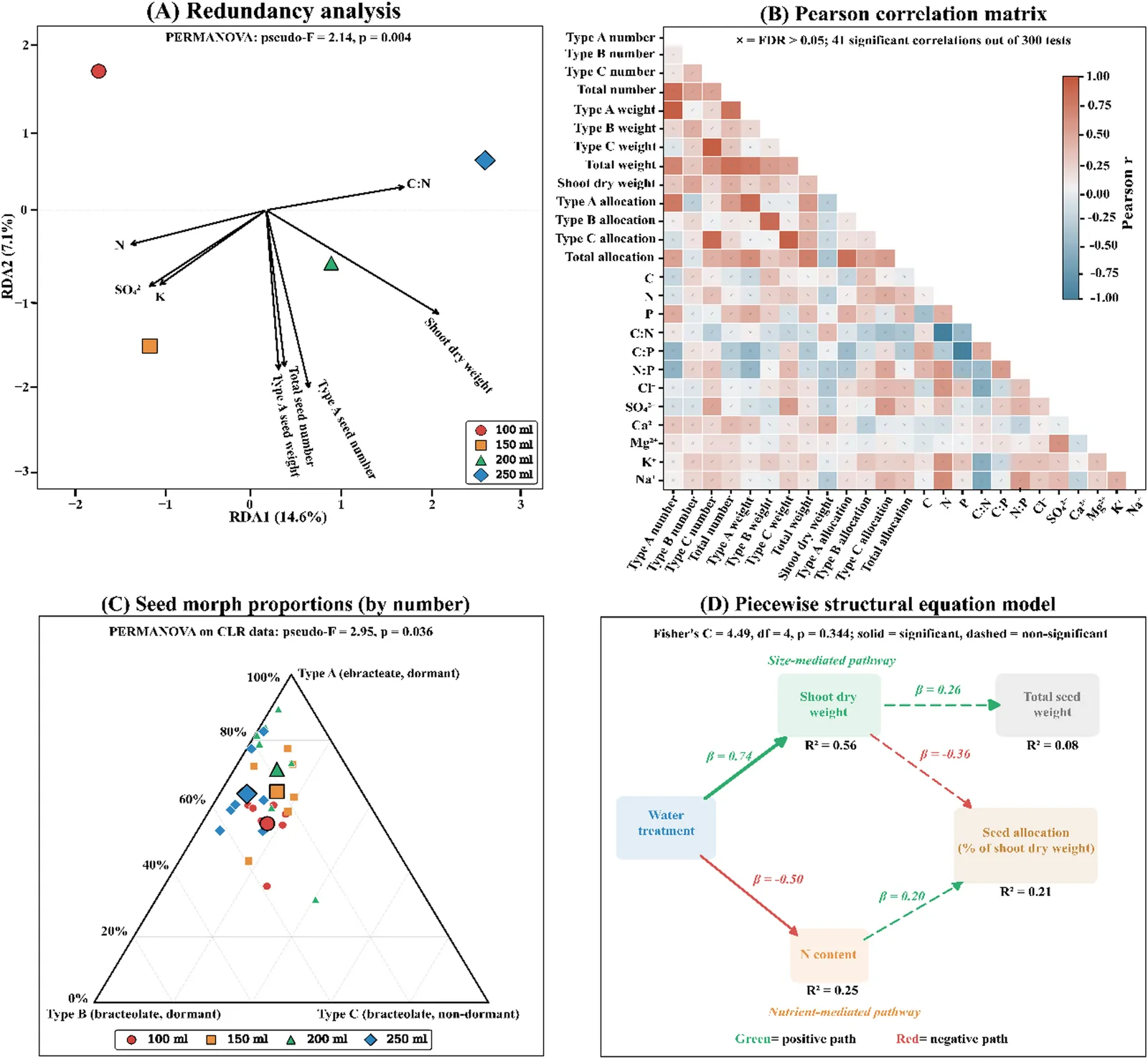
Multivariate integration of growth, reproductive, and chemical responses of *Atriplex aucheri* to water treatment. **A **Partial redundancy analysis (RDA) triplot conditioned on block effects; small symbols represent individual samples (*n* = 7 per treatment), large symbols indicate treatment centroids, and arrows show the eight strongest variable loadings on the first two constrained axes; PERMANOVA *p* value is indicated. **B **Pearson correlation heatmap of all 25 response variables computed on block-residualized data; × marks indicate non-significant correlations after Benjamini–Hochberg false discovery rate correction (*p* > 0.05); 41 of 300 pairwise tests were significant. **C **Ternary diagram showing the proportional contribution of type A, type B, and type C seeds (by number) to total seed production; each point represents one plant, large symbols indicate treatment centroids; PERMANOVA on centered log-ratio transformed proportions tested for compositional shifts among treatments. **D **Piecewise structural equation model with two hypothesized pathways: size-mediated (from water through shoot dry weight to seed allocation) and nutrient-mediated (from water through N content to seed allocation); arrow width is proportional to the absolute standardized path coefficient (β); solid arrows indicate *p* < 0.05, dashed arrows indicate *p* > 0.05; Fisher’s C statistic and *R*^*2*^ values are shown for each endogenous variable

Partial redundancy analysis, conditioned on block effects, revealed that water treatment explained 14.6% and 7.1% of the total multivariate variation on the first two constrained axes, respectively (Fig. 7a). PERMANOVA confirmed a significant overall treatment effect on the 25-variable response matrix (pseudo-*F* = 2.14, *p* = 0.004). The treatment centroids separated clearly along RDA1, with 100 mL positioned in the upper-left quadrant and 250 mL in the upper-right, indicating a gradient of increasing vegetative growth and declining nutrient concentrations with water supply. The strongest loadings on RDA1 were shoot dry weight, type A seed number, type A seed weight, and total seed number, all oriented toward higher water treatments, while N, K^+^, and SO_4_^2−^ loaded in the opposite direction.

Pearson correlation analysis with FDR correction identified 41 significant correlations out of 300 pairwise tests (Fig. 7b). Shoot dry weight was positively correlated with type A seed number and total seed number, and negatively correlated with N content, N:P ratio, and total seed allocation. The C: N ratio showed strong positive correlations with shoot dry weight and negative correlations with N content. Among ions, Na^+^ showed limited correlation with other variables.

Ternary analysis of seed morph proportions by number revealed a compositional shift across water treatments (Fig. 7c). PERMANOVA on CLR-transformed proportions confirmed a significant treatment effect (pseudo-*F* = 2.95, *p* = 0.036). The 100 mL treatment centroids were positioned with approximately equal contributions from type B and type C seeds, whereas the 250 mL centroids shifted toward higher type A and type B proportions at the expense of type C.

The piecewise structural equation model provided adequate fit to the data (Fisher’s C = 4.49, df = 4, *p* = 0.344; Fig. 7d). The model was more consistent with a size-mediated pathway than with a nitrogen-mediated pathway, although the path from shoot dry weight to seed allocation was not statistically significant; water treatment had a strong positive direct effect on shoot dry weight (β = 0.74, *p* < 0.001, *R*^2^ = 0.56), which in turn showed a negative direct effect on seed allocation (β = −0.36, *p* = 0.076) and a positive but non-significant effect on total seed weight (β = 0.26, *p* = 0.195). Through the nutrient-mediated pathway, water treatment had a significant negative direct effect on N content (β = −0.50, *p* = 0.008, *R*^2^ = 0.25), but the subsequent path from N content to seed allocation was weak and non-significant (β = 0.20, *p* = 0.304). The joint model for seed allocation explained 21% of its variance (*R*^2^ = 0.21), with shoot dry weight contributing a larger standardized effect than N content after controlling for confounding between the two predictors.

## Discussion

### Excess water promotes vegetative growth but does not enhance reproductive output

Increasing water availability in *A. aucheri* stimulated vegetative growth without producing a proportional increase in reproductive output. Across the water gradient, shoot dry weight increased by 60.6% from the 100 mL to the 250 mL day^− 1^ treatment, whereas total seed yield and total seed allocation did not increase correspondingly. This pattern indicates that excess water uncoupled biomass production from reproductive investment, consistent with life-history theory predicting that annual plants in resource-limited environments may preferentially allocate newly available resources to vegetative growth rather than proportionally increasing seed production when conditions improve [16, 17, 61]. Similar responses have been reported in other dryland and water-limited systems. In an Inner Mongolia steppe, water addition increased total biomass of dominant species while reproductive allocation declined or remained unchanged [16], and across 22 *Artemisia* species the ratio of reproductive to vegetative mass decreased with increasing soil water capacity [17]. Our results extend these observations to a heterocarpic desert annual that produces the three heteromorphic seed types [29, 34, 42]. The persistence of the growth–reproduction trade-off in a species adapted to extreme aridity suggests that this pattern operates even within diversified bet-hedging systems.

Although the categorical treatment effect on total seed allocation was not significant, allocation declined significantly across the graded water treatments according to the linear contrast (*p* = 0.022), from 10.0% at 100 mL to 6.5% at 250 mL. Under the natural habitat conditions of *A. aucheri* in the Gobi desert and arid valleys, where annual precipitation is low and evaporative demand is high [41], water is the primary limiting resource. The comparatively high reproductive allocation under the lowest water treatment may therefore reflect an adaptive strategy that maximizes seed output under conditions closer to the species’ native environment. This interpretation is consistent with previous work showing that plants derived from low-water maternal environments maintained higher seed allocation than those from high-water parents under drought conditions [34, 42]. The structural equation model further clarified the mechanism underlying this pattern. Water had a strong positive direct effect on shoot dry weight (β = 0.74, *p* < 0.001), whereas shoot dry weight showed a negative effect on seed allocation (β = −0.36, *p* = 0.076), indicating a size-mediated trade-off in which enhanced vegetative growth was not matched by proportional reproductive investment [13, 14]. Although the standardized effect of shoot dry weight on seed allocation (β = −0.36, *p* = 0.076) was larger in magnitude than that of shoot N content (β = 0.20, *p* = 0.304), neither pathway to seed allocation was statistically significant. The model was therefore more consistent with a size-mediated pathway than with a nitrogen-mediated pathway, but this interpretation remains tentative. This interpretation was reinforced by the RDA (Fig. 7A), in which shoot dry weight and seed allocation loaded in opposing directions, indicating that the growth–reproduction trade-off was a principal axis of treatment response across the water gradient.

### Differential responses of heteromorphic seed types to water availability

A distinctive feature of *A. aucheri* is the production of the three seed morphs described above ("Study species and seed collection" section) [34, 42]. The present study revealed that the three seed types responded differently to water treatment. The categorical treatment effects on the individual seed-type variables were not significant; however, polynomial contrasts detected a significant quadratic response in type A seed number and a significant linear decline in type C allocation, indicating morph-specific responses across the graded water treatments. Type A seeds showed a marginally non-significant increase in number under intermediate water (150 mL; *p* = 0.051), while type B seeds remained remarkably insensitive to water level, and type C seeds exhibited a declining allocation trend with increasing water. This differential responsiveness is consistent with the view that seed heteromorphism represents a diversified bet-hedging strategy that buffers reproductive success against environmental unpredictability, that is, spreading reproductive risk across the three seed morphs rather than between vegetative and reproductive functions [18, 19, 21].

A parallel pattern has been documented in the congener *A. sagittata*, where Mandák et al. [23] showed that type A seed production increased under favorable nutrient conditions while type C seeds contributed the majority of total fecundity and were less responsive to environmental variation. Wang et al. [24] reported that the three diaspore types of the trimorphic halophyte *A. centralasiatica* exhibited divergent germination and persistence strategies in salt desert environments, with the dormant morph providing a persistent soil seedbank and the non-dormant morph enabling rapid establishment. Wang et al. [25] further demonstrated in a field study that soil salinity regulated seedbank dynamics of the three morph types differently. The insensitivity of type B seeds to water treatment in the present study suggests that this morph may serve as the “conservative” component of the bet-hedging strategy, maintaining a stable reproductive baseline regardless of environmental conditions [29, 62].

The declining allocation to type C seeds (non-dormant, fast-germinating) with increasing water is particularly noteworthy from an ecological perspective. Under the lowest water supply, plants allocated a greater proportion of resources to type C, the morph capable of immediate germination when moisture is available. Conversely, under high water availability, the reduced investment in type C seeds and maintained production of dormant types A and B may function as a risk-spreading mechanism, ensuring that some offspring enter the soil seedbank as insurance against future drought episodes, although offspring establishment and seedbank performance were not measured here and this adaptive interpretation remains provisional [26, 63, 64]. The observation that none of the individual seed-type variables reached statistical significance under the RCBD model, despite consistent biological trends, may reflect the limited statistical power inherent in a design with seven blocks and four treatments (18 residual df). Wang et al. [34] similarly found that water treatment did not significantly alter morph allocation ratios in a binary water design, suggesting that the proportional allocation among the three seed types is relatively canalized in this species.

### Nitrogen dilution and stoichiometric shifts under increased water availability

Shoot N content showed a significant overall response to water treatment (*p* < 0.05), declining from the 150 mL to the 200 mL and 250 mL treatments, while C content remained unchanged across all water levels. This pattern resulted in a corresponding increase in the C: N ratio (*p* < 0.05). For both variables, pairwise comparisons separated the 150 mL treatment from the 200 and 250 mL treatments (Fisher’s protected LSD, *p* < 0.05), with the 100 mL treatment intermediate in both cases. The pattern identifies a threshold between intermediate and high water supply. The most parsimonious explanation for this shift is the nitrogen dilution effect, whereby biomass increases under improved water conditions without a proportional increase in N uptake, causing the concentration of N per unit biomass to decline [32, 65]. A recent global meta-analysis by Li et al. [33], encompassing 827 observations across 235 field studies, confirmed that increased precipitation consistently raises plant C: N ratios, a result directly consistent with the present findings.

Similar stoichiometric responses to graded water availability have been documented in greenhouse experiments with *Cunninghamia lanceolata*, where drought altered C: N, C: P, and N: P ratios across organs [66]. The nitrogen dilution effect has been reported in several desert and arid-zone species. Wang et al. [34] found that halophytes in arid saline environments of northwest China had elemental compositions linked to their growth strategies, with faster-growing species showing lower tissue N concentrations. The stable C content across treatments in the present study (range: 376–380 g kg^− 1^) further supports a dilution rather than an active metabolic shift, since C is the structural backbone of plant biomass and is less sensitive to short-term resource fluctuations [67].

The nutrient-mediated pathway in the structural equation model (Fig. 7d) quantified this relationship; water had a significant negative effect on N content (β = −0.50, *p* = 0.008), confirming the dilution mechanism, but the downstream path from N content to seed allocation was weak (β = 0.20, *p* = 0.304). This suggests that nitrogen dilution under excess water, while a measurable physiological shift, does not independently drive the observed changes in reproductive allocation. The correlation heatmap (Fig. 7b) reinforced this conclusion, showing that N content was negatively correlated with shoot dry weight but only weakly associated with seed allocation variables after FDR correction.

Beyond the C: N response, the N: P ratio also shifted with water supply. It declined from 1.77 at 100 mL to 1.32 at 200 mL (*p* < 0.05 for overall ANOVA), suggesting a shift toward stronger N limitation relative to P under higher water availability. Pairwise comparisons confirmed that the 100 mL treatment had a significantly higher N: P ratio than the 200 and 250 mL treatments (*p* < 0.05), with the 150 mL treatment intermediate. Water supply drives plant N: P in other water-limited systems. Precipitation addition altered plant and soil N: P in semi-arid steppe [68], and water input shifted N: P allocation in plants of semi-arid soda-alkali wetlands [69]. According to the stoichiometric framework established by Güsewell [67], N:P ratios below 14 generally indicate N limitation, a condition that applied across all treatments in the present study. The low N: P values observed here are consistent with those reported for halophytes in arid northwestern China [34] and may reflect the nutrient-poor conditions typical of desert soils in the Junggar Basin. Comparable P- or N-limited stoichiometric patterns have been reported for desert plant communities in northwest China [70, 71]. Several stoichiometric and ion variables also showed clear linear or quadratic responses to the water gradient (Table 1), consistent with the coordinated adjustment of tissue chemistry alongside growth and reproduction.

### Ion homeostasis and Na^+^ dominance in shoots

Across all water treatments, Na^+^ remained the dominant shoot cation ("Shoot ion content" section), followed by K^+^ (32–35%). This dominance persisted regardless of water level, indicating that Na^+^ accumulation is a constitutive feature of *A. aucheri* rather than a stress-induced response. High Na^+^ accumulation is a hallmark of euhalophytic species in the Chenopodiaceae/Amaranthaceae, where Na^+^ is sequestered in vacuoles and salt bladders to drive osmotic adjustment [36, 37, 72, 73]. In the closely related *A. canescens*, Na^+^ contribution to leaf osmotic potential increased substantially under both salinity and drought stress [37, 74], and salt bladder cells played a central role in maintaining ion homeostasis.

Among the six ions analyzed, only SO_4_^2−^ content was significantly affected by water treatment (*p* < 0.05), declining by 45.9% from the 150 mL to the 250 mL treatment. Glenn et al. [75], working with three Atriplex species, reported that sulfate concentrations decreased with increasing salinity, and a similar dilution mechanism may operate under increased water supply. Reginato et al. [76] reviewed the differential effects of SO_4_^2−^ versus Cl^−^ on halophytes, noting that sulfate dynamics are regulated by active transport mechanisms distinct from those controlling chloride uptake. The declining SO_4_^2−^ content under higher water in the present study may reflect increased leaching from the root zone or dilution within expanding shoot tissue, though the exact mechanism requires further investigation.

The non-significant declining trends in Cl^−^, Na^+^, and K^+^ contents under increasing water are consistent with a general dilution effect similar to that observed for N. Maintained K^+^/Na^+^ homeostasis has similarly been reported in highland quinoa cultivars across a graded salinity gradient, where leaf K^+^/Na^+^ ratio served as the primary indicator of salt tolerance [77]. Nada et al. [78] demonstrated that Na^+^ plays a vital physiological role in the xero-halophyte *A. halimus* during drought recovery, supporting the interpretation that the high and stable Na^+^ levels in *A. aucheri* shoots reflect a constitutive osmoregulatory strategy rather than passive uptake.

### Limitations and future directions

Several limitations of the present study should be acknowledged. The experiment used pot-grown plants in a greenhouse, which may not fully capture natural desert conditions where plants experience fluctuating water availability, competition, and edaphic heterogeneity. Water was supplied as fixed daily volumes rather than the episodic pulses that characterize arid precipitation, and heteromorphic seed production may respond differently under pulsed versus continuous water supply. The sample size (*n* = 7 blocks) limited power to detect moderate effects, particularly for seed allocation variables where biological trends were consistent but non-significant. Only the F1 generation was examined; the transgenerational effects of water-induced shifts in seed ratio and allocation on F2 performance were addressed in our companion study [42] under a different experimental framework. Because all four treatments exceeded the minimum water level required for survival, the design represents a gradient from baseline to excess supply rather than a stress-versus-control comparison, with 100 mL day^− 1^ serving as the reference treatment for all pairwise comparisons. Given the modest sample size (*n* = 28 plants), the piecewise structural equation model was kept simple, with few estimated paths. It was used as a hypothesis-guided assessment of two pre-specified competing pathways rather than an exhaustive causal model; therefore, the pathway estimates should be considered indicative and require confirmation with larger datasets. Finally, only aboveground responses were quantified; root biomass, architecture, and health were not assessed, so belowground treatment effects cannot be ruled out, although no obvious aboveground symptoms of severe waterlogging were observed.

Future research should address these limitations through field experiments under natural rainfall gradients across the Junggar Basin and Gobi desert, which would test whether the growth–reproduction trade-off observed under controlled conditions persists under natural environmental variability. Increasing the number of water treatment levels and replicates would provide greater statistical power and enable dose–response modeling. In particular, future work should measure belowground traits. Pairing root data with the heteromorphic seed and stoichiometric responses examined here would show how this desert halophyte allocates resources across the whole plant.

## Conclusion

Our results show that the effects of increasing water availability on reproductive investment in *Atriplex aucheri* were more consistent with a size-mediated growth response than with a nutrient-mediated pathway, although the size-mediated relationship should be regarded as tentative because the path from shoot dry weight to seed allocation was not statistically significant. Although higher water supply substantially increased shoot biomass, this increase was not matched by proportional gains in seed yield or total seed allocation, indicating a decoupling of vegetative growth from reproduction. The three seed morphs showed distinct response patterns, with type A tending to increase at intermediate water, type B remaining comparatively stable, and type C allocation declining under higher water availability, consistent with differential roles in the species’ bet-hedging strategy. Increased water also reduced shoot nitrogen concentration and increased the C: N ratio, supporting a nitrogen dilution effect associated with rapid biomass accumulation, while sodium remained the dominant cation across treatments, indicating relatively stable concentrations of most measured shoot ions across the tested water treatments. Together, these findings indicate that excess water reshapes the growth-reproduction balance and stoichiometric status of this desert annual, while most measured shoot ions remain relatively stable, providing species-specific insight into how *Atriplex aucheri* responds to graded water supply under greenhouse conditions.

## Supplementary Information

Supplementary Material 1.

## Data Availability

The datasets used and/or analyzed during the current study are available from the corresponding author upon reasonable request.
