# Supplementary material for: Graded water supply decouples vegetative growth, reproductive investment, and nitrogen balance in trimorphic Atriplex aucheri
Source: BMC Plant Biol. 2026 Aug 3;26:1857. doi: 10.1186/s12870-026-09676-3 (PMC13721675; doi:10.1186/s12870-026-09676-3)
Supplement: Supplementary file 1 [file 12870_2026_9676_MOESM1_ESM.docx]

**Supplementary Information**

**Supplementary figure**

**Fig. S1.** Relative seed-morph composition of *Atriplex aucheri* across four water treatments. (a) Composition by number, showing each seed morph (type A, type B, type C) as a percentage of the total number of seeds produced per plant. (b) Composition by yield, showing each morph as a percentage of total seed mass per plant. Bars are treatment means (n = 7 blocks per treatment). The proportional contribution of each morph to total seed yield did not differ significantly among water treatments, indicating a stable yield-based seed portfolio. By number, the contribution of type B varied significantly among treatments (*p* = 0.022) and that of type C declined along a significant linear trend (*p* = 0.033). Relative composition was analyzed using the randomized complete block ANOVA model with water treatment and block as factors.
